# Molecular mechanisms underlying totipotency

**DOI:** 10.26508/lsa.202302225

**Published:** 2023-09-04

**Authors:** Takashi Ishiuchi, Mizuki Sakamoto

**Affiliations:** Faculty of Life and Environmental Sciences, University of Yamanashi , Yamanashi, Japan

## Abstract

Numerous efforts to understand pluripotency in mammals, using pluripotent stem cells in culture, have enabled the generation of artificially induced pluripotent stem cells, which serve as a valuable source for regenerative medicine and the creation of disease models. In contrast to these tremendous successes in the pluripotency field in the past few decades, our understanding of totipotency, which is highlighted by its broader plasticity than pluripotency, is still limited. This is largely attributable to the scarcity of available materials and the lack of in vitro models. However, recent technological advances have unveiled molecular features that characterize totipotent cells. Single-cell or low-input sequencing technologies allow the dissection of pre- and post-fertilization developmental processes at the molecular level with high resolution. In this review, we describe some of the key findings in understanding totipotency and discuss how totipotency is acquired at the beginning of life.

## Abstract (precis)

A review article summarizing recent findings related to totipotency in mammals, encompassing topics such as the epigenome and gene regulation in early embryos, in vitro totipotent-like cells, and somatic nuclear transfer.

## Introduction to Totipotency—How is it Defined?

Pluripotency is defined as the cellular ability to differentiate into all three germ layers of the embryo. In contrast, totipotency can be defined as the ability of a cell to contribute to all lineages in an organism (Ishiuchi & Torres-Padilla, 2013; Condic, 2014; Posfai et al, 2021). According to this definition, the zygote, formed by the fusion of an egg and a sperm, is totipotent. Although the concept of zygotes being totipotent is widely and easily accepted, when totipotency is acquired and how long totipotency is maintained during development are not entirely clear. This is because totipotency can be interpreted in different ways: it can be defined stringently as the ability of a single cell to develop into a complete organism by itself (i.e., organism formation ability) or less stringently as the ability to differentiate into all lineages. To distinguish these differences, it has been proposed to call the former “totipotency” and the latter “plenipotency” (Condic, 2014). Though the term plenipotency is not yet commonly used, this terminology underscores the importance of carefully using the term totipotency, especially in the stem cell research field where the induction of “totipotent-like” cells in culture has been recently actively studied.

In stem cell research, totipotent-like cells often refer to cells with broader plasticity than pluripotent stem cells. As mouse embryonic stem cells (mESCs) have limited differentiation capacity toward extraembryonic lineages, mouse cells that can differentiate into both embryonic and extraembryonic lineages are generally recognized as totipotent-like (Posfai et al, 2021). However, human embryonic stem cells (hESCs) exhibit considerably different characteristics. Primed hESCs, similar to late post-implantation epiblast cells, can differentiate into trophoblasts upon BMP4 stimulation (Xu et al, 2002). Moreover, naïve hESCs, which resemble pre-implantation epiblast cells (Semi & Takashima, 2021), can differentiate into trophoblast stem-like cells by simply changing the medium composition (Cinkornpumin et al, 2020; Dong et al, 2020; Guo et al, 2021; Io et al, 2021; Kobayashi et al, 2022). Notably, naïve hESCs by themselves form blastocyst-like structures constituted of the inner cell mass and trophectoderm, called blastoids, by being aggregated and cultured in a specific condition that contains ERK and Nodal signaling inhibitors (Fan et al, 2021; Liu et al, 2021; Sozen et al, 2021; Yanagida et al, 2021; Yu et al, 2021; Kagawa et al, 2022), highlighting the broader plasticity of naïve hESCs compared with that of mESC. Recent studies also generated post-implantation embryo-like structures containing both embryonic and extra-embryonic lineages from naïve hESCs through alterations in the medium composition or via transcription factor-mediated lineage specification (Ai et al, 2023; Liu et al, 2023; Pedroza et al, 2023; Weatherbee et al, 2023). Although post-implantation embryo-like structures could be established from mESCs, the forced expression of specific transcription factors to promote lineage conversion is essential (Lau et al, 2022; Tarazi et al, 2022). These findings on the intrinsic capacity of hESCs raise the question of whether mouse cells with the ability to differentiate into both embryonic and extraembryonic lineages should be recognized as totipotent-like cells. In this sense, the term “totipotent-like” would be confusing. Given that totipotency refers to the cell potency of an embryo, it is important to investigate which embryo characteristics are captured in vitro and whether and how the shared characteristics relate to cell potency.

### Gene expression profile in totipotent cells

**Figure 1.**
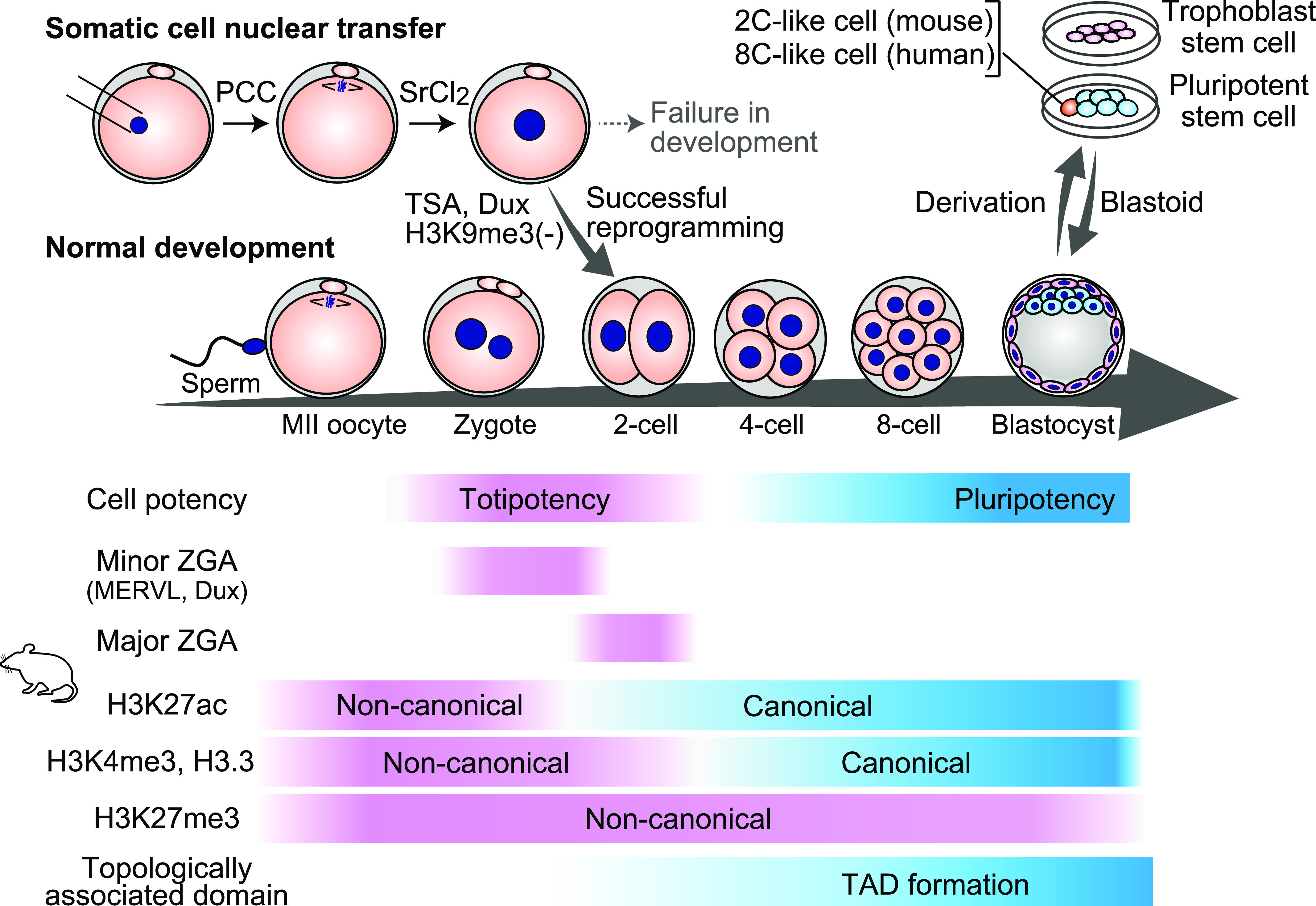
Totipotency in development. A schematic illustration of mouse embryonic development upon fertilization is shown and denoted by “normal development.” In this developmental process, dynamic changes in cell potency, transcription, and epigenetic status have been observed, but their interdependencies remain to be understood. Somatic cell nuclear transfer represents a unique technology to artificially induce a totipotent cell, providing an opportunity to assess efficiencies in acquiring totipotency upon experimental manipulation. 2C-like cells or eight-cell-like cells found in pluripotent stem cell cultures also serve as experimental tools for analyzing the molecular mechanisms regulating early embryonic cell identity.

The transcriptome of totipotent zygotes shows a profile highly similar to that of unfertilized eggs (Hamatani et al, 2004; Xue et al, 2013; Yan et al, 2013; Deng et al, 2014). This is because the zygote inherits most mRNAs accumulated in the egg. Such inherited mRNAs, called maternal mRNAs, are diluted and degraded during postfertilization development. Thus, embryos must initiate transcription from their own genome to newly synthesize mRNAs and proteins necessary for embryonic development. This de novo transcriptional activation after fertilization is called zygotic genome activation (ZGA), and ZGA is considered a gene expression program unique to the early embryo. In mice, ZGA is observed from the late one-cell stage after fertilization; ZGA from the late one-cell stage to the early two-cell stage is weak in transcriptional activation and is called minor ZGA (Bouniol et al, 1995; Eckersley-Maslin et al, 2018; Svoboda, 2018). In contrast, strong transcriptional activation occurs from the mid-to-late two-cell stage and is called major ZGA (Fig 1). To distinguish their biological significance, transcription at minor ZGA and major ZGA was inhibited by a reversible transcription inhibitor (Abe et al, 2018). The results showed that development to the blastocyst stage is generally normal when major ZGA is inhibited, whereas developmental arrest at the two-cell stage occurs upon minor ZGA inhibition. Therefore, temporally regulated transcription initiation and/or the gene products from minor ZGA might be important in the totipotency window. However, the results should be interpreted carefully, as the inhibition of minor ZGA also affects major ZGA as well (Abe et al, 2018; Liu et al, 2020). The question is whether and how ZGA is linked to the acquisition of totipotency. This question is important but difficult to answer because failures in ZGA, normally associated with developmental phenotypes, do not necessarily mean a loss of totipotency. A comprehensive understanding of the molecular basis of ZGA would allow us to reconstitute the ZGA process and test whether its reconstitution alters cell potency. In any case, clarifying how ZGA is regulated is of biological importance, as it gives us the basic knowledge of how genes initially control our life.

### In vitro models shed light on the molecular mechanisms regulating ZGA

Macfarlan et al identified two-cell-like cells (2CLCs) that express the MERVL retrotransposon and other two-cell-specific transcripts as a minority subpopulation in mESC cultures (Macfarlan et al, 2012). A similar subpopulation of mESCs was previously identified by Zscan4 expression (Falco et al, 2007), yet 2CLCs constitute a subpopulation of Zscan4-positive cells (Eckersley-Maslin et al, 2016; Rodriguez-Terrones et al, 2018). 2CLCs show differentiation capacity toward both embryonic and extraembryonic lineages; therefore, 2CLCs are often referred to as totipotent-like cells. Although their global similarity with two-cell embryos appears to be limited (Kolodziejczyk et al, 2015), the identification of 2CLCs has enabled the experimental dissection of how ZGA is regulated in vitro. Several epigenetic and chromatin regulators regulate two-cell-like signatures (Baker & Pera, 2018; Genet & Torres-Padilla, 2020; Iturbide & Torres-Padilla, 2020). For example, trichostatin A (TSA), a histone deacetylase (HDAC) inhibitor, increased the 2CLC population (Macfarlan et al, 2012), and other negative regulators of 2CLC emergence, including the CAF-1, PRC1.6, and EP400-Tip60 complexes, have been identified (Ishiuchi et al, 2015; Rodriguez-Terrones et al, 2018). Most importantly, Dux, a homeobox transcription factor, has been identified as a molecular trigger for the emergence of 2CLCs (De Iaco et al, 2017; Hendrickson et al, 2017; Whiddon et al, 2017). Dux overexpression in mESCs robustly induced 2CLCs. Moreover, the depletion of Dux completely abolished the emergence of 2CLCs. Even with this accumulating knowledge on 2CLCs, why 2CLCs spontaneously arise in mESC culture is still an open question. In vivo, Dux is expressed in zygotes and two-cell mouse embryos, suggesting that Dux is a key regulator of ZGA. However, Dux knockout mice were able to develop into adulthood despite showing impaired expression of Dux target genes, including MERVL, delay in pre-implantation development, and reduced litter size (Chen & Zhang, 2019; De Iaco et al, 2020). Thus, other key factors may compensate for Dux activity to regulate ZGA in vivo. This scenario sounds reasonable, as the acquisition of totipotency is the most critical point in life and should be protected by several backup mechanisms.

In humans, ZGA occurs around the four-cell and eight-cell stages. Recently, similar to the concept of 2CLCs, eight-cell-like cells (8CLCs) were identified in naïve hESC cultures (Taubenschmid-Stowers et al, 2022). DUX4, the human ortholog of mouse Dux, also promoted 8CLC signatures, suggesting a conserved role of Dux in ZGA. In contrast to the conserved activity of DUX4, the TPRX homeobox transcription factor family, which is not well conserved in mice (Maeso et al, 2016), and DPPA3 (also known as STELLA) have been identified as key regulators for the emergence of 8CLCs and ZGA in human embryos (Mazid et al, 2022; Zou et al, 2022).

In contrast to the transcription mode in other cell types, the positional selection of transcription start sites is ambiguous in minor ZGA. In addition, splicing activity is extremely low during minor ZGA (Abe et al, 2015). Related to these findings, spliceosome inhibition in mESCs promoted the expression of two-cell embryo-specific genes and conferred differentiation ability toward both embryonic and extraembryonic lineages (Rodriguez-Terrones et al, 2018; Shen et al, 2021). These cells, induced by the spliceosome inhibitor pladienolide B (PlaB), are termed totipotent blastomere-like cells. Interestingly, treatment of hESCs with PlaB also up-regulated ZGA-related genes, suggesting a conserved role of spliceosome regulation for ZGA. The precise mechanism by which totipotent blastomere-like cells are formed, including the role of Dux, should be determined to understand the molecular basis underlying the altered cell potency.

### Epigenetic features of totipotent embryos

Early mouse embryos displayed unique epigenetic landscapes that were largely different from those of other cell types, which led to the hypothesis that they might be required for totipotency. Recent technical advances have greatly facilitated the understanding of epigenetic status in early embryos (Fig 1). For example, using a low-input ChIP-seq technique, it has been shown that H3K4me3 in mouse zygotes forms atypical broad domains (Dahl et al, 2016; Liu et al, 2016; Zhang et al, 2016). The noncanonical form of H3K4me3 (ncH3K4me3) is reprogrammed in two-cell embryos, and sharp H3K4me3 peaks typically emerge in late two-cell embryos. Transcription inhibition and depletion of KDM5A and KDM5B suppressed H3K4me3 reprogramming, indicating that KDM5A and KDM5B expressed de novo after fertilization are required. H3.3, a histone H3 variant, is generally enriched in active chromatin regions; however, H3.3 is atypically uniformly distributed across the genome in zygotes (Ishiuchi et al, 2021). Analogous to H3K4me3 reprogramming, ncH3.3 transitions to the canonical form at the two-cell stage, and H3.3 becomes enriched at the active chromatin regions at the late two-cell stage. In the case of H3.3, DNA replication rather than transcription is required for the transition, indicating that the reprogramming of H3K4me3 and H3.3 distribution are independently regulated. H3K27ac, another active chromatin mark, also forms unusually broad domains in zygotes, and its distribution transitions to a canonical pattern (Wang et al, 2022). However, its reprogramming kinetics is different; H3K27ac formed sharp peaks already at the early two-cell stage, preceding the reprogramming of H3K4me3 and H3.3. Although the underlying mechanisms of H3K27ac reprogramming are yet to be clarified, the finely balanced activity between HDAC and p300/CBP in the local chromatin environment might be responsible for the quick changes in H3K27ac enrichment. A notable difference has been observed in the distribution of H3K27me3, a repressive chromatin mark, as its noncanonical broad domains were maintained throughout pre-implantation development (Zheng et al, 2016). In parallel with these dynamic changes in histone modifications, topologically associated domains are relatively slowly and gradually established during pre-implantation development (Du et al, 2017; Ke et al, 2017). Given these results, it would be intriguing to address how enhancer–promoter interaction is regulated during early development, as it has been suggested that the transition from enhancer-independent to enhancer-dependent transcription occurs during ZGA (Majumder et al, 1993).

DNA methylation status in early embryos has also been extensively investigated (Seisenberger et al, 2013; Smith & Meissner, 2013; Stewart et al, 2016; Greenberg & Bourc’his, 2019). The paternal genome undergoes active DNA demethylation, whereas the maternal genome is relatively hypomethylated in zygotes. These findings give rise to the idea that the DNA methylation status might be crucial for acquiring totipotency. However, the nuclear transfer of the DNA methylation-free genome into enucleated oocytes allowed pre-implantation development (Sakaue et al, 2010). This suggests that the presence of DNA methylation might not be necessary for the initial phase of early development although genomic imprinting through DNA methylation is required later in development. Conversely, the absence of Stella (also known as Dppa3) in oocytes and zygotes led to DNA hypermethylation and impaired pre-implantation development (Li et al, 2018; Han et al, 2019). Although defects in the cytoplasm have also been suggested in Stella mutant embryos, these results might suggest that a DNA-hypomethylated status is preferred for the acquisition of totipotency.

One of the epigenetic features of totipotent zygotes is the inheritance of epigenetic marks from gametes. As a result, the zygote is characterized by the establishment of an asymmetric epigenome between male and female pronuclei (Burton & Torres-Padilla, 2014). These chromatin asymmetries, at least partially, reflect the differences in chromatin composition acquired during gametogenesis. During oogenesis, oocyte-specific histone modifications and DNA methylation patterns are gradually established (Sendžikaitė & Kelsey, 2019; Inoue, 2023). H3K27me3 forms unusual broad domains during oogenesis. H3K36 methylation likely plays a key role in the establishment of the epigenome in oocytes, as the antagonism between H3K27me3 and H3K36 methylation has been well documented (Schmitges et al, 2011; Yuan et al, 2011). Indeed, H3K27me3 distribution was altered in the absence of H3K36me3 in mouse oocytes (Xu et al, 2019). Although DNA methylation is also suggested to antagonize H3K27me3 (Holoch & Margueron, 2017), this seems to be not the case in mouse oocytes, as H3K36me3 and H3K27me3 patterns were unaffected in the absence of DNA methylation (Xu et al, 2019). DNA methylation is likely downstream of H3K36me2/me3 (Xu et al, 2019; Yano et al, 2022). Interestingly, it has been shown that maternal H3K27me3 inherited by zygotes and early embryos contributes to the suppression of gene expression from the maternal allele (Inoue et al, 2017). This serves as a DNA methylation-independent noncanonical imprinting mechanism, which is required for imprinted X inactivation and normal placental development in mice.

Whereas maternal epigenetic status tends to be inherited by the zygote, paternal chromatin undergoes extensive remodeling upon fertilization. This is because sperm protamines incorporated during spermiogenesis need to be replaced by histones after fertilization (Rathke et al, 2014). However, a small percentage of histones are retained in mature mammalian sperm (Hammoud et al, 2009; Brykczynska et al, 2010). The retained histones in mouse sperm are mainly located in CpG-rich regions, especially at promoters, and are composed of H3.3 modified with active marks H3K27ac, H3K9ac, and H3K4me3 (Erkek et al, 2013; Jung et al, 2017; Yoshida et al, 2018). Some promoters were marked by both H3K4me3 and H3K27me3, suggesting the presence of bivalent promoters in the sperm. In contrast to this view, other groups have shown that nucleosomes are located at the gene desert regions in sperm (Carone et al, 2014; Yamaguchi et al, 2018), suggesting that the determination of nucleosome-retained genomic regions might need further careful investigation. The establishment of the sperm-specific epigenetic pattern seems to be important as the injection of haploid round spermatids into oocytes results in poor development, possibly because of abnormal inheritance of epigenetic information from immature sperm (Ogura et al, 1994; Kishigami et al, 2006b; Sakamoto et al, 2022). The presence of modified histones in sperm suggests that they may regulate post-fertilization embryo development. Indeed, alterations in H3K4me2/3 levels in mouse sperm affect postnatal development (Siklenka et al, 2015; Lismer et al, 2021). Thus, the epigenetic features acquired during gametogenesis are important for shaping the epigenome supporting totipotency.

### Somatic cell nuclear transfer (SCNT)

SCNT, which involves the injection of a somatic nucleus into an enucleated oocyte, gives rise to a complete organism in several species (Ogura et al, 2021). Thus, SCNT represents the best example for experimentally inducing a totipotent cell. SCNT revealed that the ooplasm can reprogram somatic nuclei or the epigenome to a totipotent state, although the underlying mechanism is still unclear. To address the reprogramming mechanism, one can perform a knockdown experiment to deplete the oocyte factor of interest and observe its outcome. However, even if there were any developmental phenotypes, it is difficult to say that the factor is required to acquire totipotency. Thus, an experimental approach to facilitate totipotency acquisition is important to gain insight into its underlying mechanism. Fortunately, or unfortunately, the success rate of SCNT is still limited, and therefore, it provides an opportunity to design such an experiment.

SCNT embryos are generated in several steps (Matoba & Zhang, 2018) (Fig 1). First, somatic nuclei are injected into enucleated oocytes at the metaphase II stage. The injected somatic nuclei then undergo premature chromosome condensation (PCC) and form metaphase-like chromosomes. Reconstructed SCNT embryos initiate embryonic development upon activation by reagents such as strontium and then form pseudo-pronuclei, which highly resemble pronuclei formed in normal zygotes. Whereas a normal zygote forms two pronuclei, constituted of maternal and paternal genomes, an SCNT embryo normally forms one or two pseudo-pronuclei, depending on the distribution of PCC chromosomes. Because SCNT embryos are formed through these steps, each process may be involved in totipotency acquisition. Treatment with TSA, an HDAC inhibitor, during and immediately after the activation step improves the success rate of SCNT embryo development (Kishigami et al, 2006a). Thus, it is likely that increasing global histone acetylation levels during the PCC or pseudo-pronuclear formation process helps to lower the barrier toward the acquisition of totipotency. Although TSA showed a mild effect on the transcriptome of two-cell SCNT embryos (Inoue et al, 2015), it greatly restored H3K9ac levels at aberrantly high or low acetylated regions in SCNT embryos (Yang et al, 2021). Matoba et al identified genomic regions that failed to be transcriptionally activated during ZGA in SCNT embryos (Matoba et al, 2014). These regions, referred to as reprogramming-resistant regions, are highly enriched in H3K9me3 in donor cells. Intriguingly, H3K9me3 removal by Kdm4d mRNA injection considerably improved SCNT embryo development. Although both TSA and H3K9me3 removal improve SCNT embryo development, transcription from reprogramming-resistant regions is rescued by H3K9me3 removal but not by TSA (Yang et al, 2021), suggesting that these two approaches improve SCNT embryo development through different mechanisms. Further investigation revealed that correction of down-regulated Dux expression in SCNT embryos is the key downstream event of these treatments, as increasing the Dux expression level highly improved the H3K9ac landscape and SCNT embryo development (Yang et al, 2020, 2021). Importantly, the effects of TSA and H3K9me3 removal were canceled when Dux-null donor cells were used. Thus, these results strongly suggest that Dux might be one of the inducers of totipotency and that epigenetic status permissive for Dux expression is important for totipotency acquisition.

### Future perspective

Experimental techniques applicable to mammalian early embryos have been limited until recently. However, recent technical advances, including single-cell or low-input sequencing technology, have made considerable progress toward understanding the molecular basis underlying mammalian early development and how much zygotes or early embryos differ from other cell types. Using these technologies, we can accumulate knowledge to describe the specific features of totipotent cells. The next question that comes up is, “What is necessary and sufficient for the acquisition of totipotency?” For this understanding, finely controlled loss-of-function experiments, such as a degron-mediated acute protein degradation system (Abuhashem et al, 2022), would be important to determine whether a molecule of interest is required during the narrow totipotency window. This is particularly important when analyzing the roles of oocyte-derived maternal factors. To perform such an experiment, it is necessary to generate knock-in mice, which is time-consuming and applicable only to a few genes. An antibody-mediated protein depletion system might serve as a quick alternative approach, although it would require improved temporal regulation, specificity, and robustness (Clift et al, 2017). Moreover, to deepen our understanding, these loss-of-function experiments should ideally be carried out on an unbiased screening platform; however, a genome-wide genetic screening platform applicable to mammalian early embryos is currently unavailable. In determining the function of each gene, its expression at both the RNA and protein levels should also be considered. RNA expression can be analyzed with high sensitivity, but proteomic analysis is relatively less sensitive. Low-input ribosome profiling techniques have enabled the capture of RNA molecules being translated in mammalian oocytes and early embryos (Luong et al, 2020; Xiong et al, 2022; Zhang et al, 2022), providing insights into the dynamics of translation during the reprogramming of cell potency. Information on totipotent cells is currently accumulating, and thus, further technical innovations are required to fully understand the molecular mechanisms underlying totipotency.
